# Phenotypic testicular abnormalities and pubertal development in boys with McCune-Albright syndrome

**DOI:** 10.1186/s13052-018-0577-7

**Published:** 2018-11-19

**Authors:** Tommaso Aversa, Giuseppina Zirilli, Domenico Corica, Filippo De Luca, Malgorzata Wasniewska

**Affiliations:** Department of Human Pathology in Adulthood and Childhood, University of Messina, Via Consolare Valeria, 98124 Messina, Italy

**Keywords:** Macroorchidism, Peripheral precocious puberty, Testicular lesions, Testicular microlithiasis, Therapeutic strategies

## Abstract

Aim of this survey is to review the few available literature data on pathophysiologic and clinical aspects of pubertal development in boys with McCune-Albright syndrome (MAS). On the basis of such analysis, we concluded that:

1) peripheral precocious puberty (PPP) is significantly more infrequent in boys than in girls; 2) the most common testicular abnormality at MAS presentation is macroorchidism, that may be either monolateral or bilateral; 3) macroorchidism is not always associated with clinical and biochemical evidence of PPP; 4) testicular microlothiasis is distinctly more frequent in boys with MAS than in those without MAS; 5) the available therapeutic schedules have to be adopted already at MAS presentation only in the cases with PPP.

## Background

McCune-Albright syndrome (MAS, OMIM 174800) is a rare disorder caused by a post-zygotic gain-of-function mutation at codon 201 in the GNAS gene [1].

MAS is classically characterized, from a clinical view, by the triad of polyostotic fibrous dysplasia, café-au-lait spots and peripheral precocious puberty (PPP).

Phenotypic expression of MAS, however, may significantly vary according to gender. In fact, while PPP is a very usual initial manifestation in girls [2], it has been reported, by contrast, in only a minority of boys [3], which might explain why this disorder is diagnosed more often and earlier in girls [2]. Furthermore, in the few reported boys with MAS and autonomous testicular early activation, pubertal development is not always characterized by the same clinical course [4–9].

Considering that a large majority of the MAS patients reported in the literature are females [10], we aim to review, in the present commentary, the few available literature data on pathophysiologic and clinical aspects of pubertal development in MAS boys and on therapeutic management of those with associated PPP. For this purpose, we performed a systematic investigation through MEDLINE via PubMed (https://www.ncbi.nlm.nih.gov/pubmed), EMBASE, Web of Science, Semantic Scholar and Cochrane Library, based on the following four key-words: McCune-Albright syndrome, precocious puberty, pubertal development and boys. We took into consideration all the original articles (5 papers), the isolated case reports (11 papers) and the systematic reviews (2 papers) on MAS-related pubertal development in boys, which were published during the period from 2000 to 2018 (references [2–19]).

### Pathophysiologic aspects of pubertal development in MAS boys

In contrast to girls, only a minority of boys with MAS exhibit a picture of PPP, whose prevalence was reported to range around 42% in the very large pediatric cohort by Boyce et al. [11], including 26 boys aged between 3 and 15 years. This prevalence is very far from the one observed in MAS girls at the Bethesda Institutes of Health, that is 83% [11].

Such a gender dimorphism in the prevalence of PPP might be explained on the basis of the same competence to produce sex steroids that is peculiar of both the ovarian cell populations (granulosa and theca cells). By contrast, the two testicular cell populations (Leydig and Sertoli cells) play a different physiologic role in the hormone biosynthesis. Therefore, whereas in MAS girls the activating mutation in the GNAS gene is almost always followed by a precocious increase of estrogen secretion, in boys an early androgen secretion may be observed only when this mutation involves Leydig cells, but not in the cases where are the Sertoli cells to carry the mutation [12].

In fact, when testicular autonomous hyperfunction is restricted to Sertoli cells, testicular enlargement has been reported to be associated with elevated serum levels of inhibin B and anti-müllerian hormone, whilst testosterone and gonadotropin serum concentrations remain in the pre-pubertal range [7]. In these cases, obviously, macroorchidism is not accompanied by signs of PPP [4, 5, 7, 8]. In contrast, when testicular autonomous hyperfunction involves Leydig cells, macroorchidism is usually associated with a clinical and hormonal picture of PPP [3, 6].

### Phenotypic testicular abnormalities in MAS boys

According to the study by Boyce et al. [11], the most common testicular abnormality at MAS presentation seems to be macroorchidism, a clinical feature that was detected in 50% of the children included in that series and was unilateral in around 15% of them. In only 42% of those boys with either monolateral or bilateral testicular enlargement, this finding was associated with other clinical findings of PPP [11].

Unilateral macroorchidism as a presenting clinical manifestation of MAS has been reported also by other authors [4–6, 13] and may be interpreted on the light of a monolateral occurrence of the mutational event. It is more frequent, however, that macroorchidism in MAS boys is bilateral and generally in the context of a PPP [3], even though a bilateral testicular enlargement has been also reported in association with autonomous hyperfunction of Sertoli cells and no signs of early puberty [5].

Another phenotypic abnormality that is known to be possibly found at testis level in MAS boys is testicular microlithiasis (TM) [7, 11, 14], i.e. an ultrasonographic (US) picture whose prevalence in pediatric general population is estimated to range around 5% [15]. Surprisingly, TM prevalence in MAS boys was reported to be distinctly higher, that is from 30 to 62% [11, 14], which suggests that the association between MAS and TM cannot be considered as an incidental finding, although the clinical implications of such association have not been clarified to now [7, 11, 12, 14].

Apart from TM, other common US findings in MAS males are focal hyper- and hypoechoic lesions, that were found in 49 and 30%, respectively, of the adult and young patients included in the overall series by Boyce et al. [11]. Other US findings in the same series included diffuse heterogeneity (47% of cases) and focal calcifications (11%). All these lesions were either bilateral (in 74% of cases) or monolateral (in the remaining 26%) and ranged from several millimeters to over four centimeters [11].

The patients with the above-described US lesions were followed up for several years and none of them developed any signs of malignant transformation [11]. Moreover, in 83% of cases no minimal progression in size of the lesions was recorded. Therefore, the authors recommended a conservative approach to management of MAS-related testicular US lesions, with emphasis to testis preservation, close clinical follow-up and serial US evaluations [11].

### Long-term prognosis of testicular function

Due to the rarity of MAS in males, data concerning the natural evolution of sexual development and the adult fertility prognosis in boys are very scanty. One of the few available studies on the long-term evolution of testicular function in a boy with MAS regards an individual, who had initially presented with a picture of unilateral macroorchidism and very precocious puberty and was subsequently followed up for more than 15 years, from 2.9 to 18 years [6]. According to the results of this prolonged follow-up, it might be argued that MAS boys seem to be able to maintain their autonomous testicular hyperfunction for a long time [6]. In fact, at the end of follow-up period, that boy exhibited an effective spermatogenesis, with the evidence of germ cell maturation in the histologic testis samples despite the persistence of an autonomous testicular hyperfunction with marked FSH suppression [6].

There is in the literature another MAS boy who was followed up for a very long period: from 4.6 to 16.6 years [16]. This additional patient showed, at the conclusion of follow-up, a complete pubertal development, with adult pubic and axillary hair and normal penile length. In that individual, who had initially presented with monolateral macroorchidism and no clinical and biochemical signs of early Leydig cell activation, a progressive development of both testes was recorded during follow-up, after the spontaneous activation of pituitary-gonadal function. However, an evident asymmetry in testicular sizes (12 vs 25 ml) persisted still at the age on 16.6 years [16].

This last case report suggests that, in a MAS boy presenting with no signs of PPP and a monolateral testicular enlargement due to isolated involvement of Sertoli cells, a consecutive activation of Leydig cells may subsequently occur as a consequence of a physiologic pituitary stimulation [16].

### Therapeutic strategies in MAS boys with abnormalities of pubertal development

In the cases with precocious macroorchidism, it should be preliminarily evaluated whether the activating mutation involves either Sertoli cells or Leydig cells or both. In fact, in the cases with isolated Sertoli cell hyperplasia and no clinical and biochemical evidence of PPP, no treatment should be started, at least initially [4, 7]. In such cases a treatment might be considered only subsequently, when a consecutive activation of Leydig cells follows the initially isolated involvement of Sertoli cells [16, 17].

By contrast, in the cases presenting with PPP and either unilateral or bilateral testicular enlargement, a pharmacological treatment should be begun very early, in order to counterbalance the effects of androgens on growth and bone maturation [6].

There are only few reports on the long-term results of pharmacological therapies in MAS boys with PPP [9, 11, 16–19], which are, furthermore, based on a limited number of patients [12]. According to these reports, therapeutic strategies should be based on the association of two kinds of drugs, aiming at both suppressing gonadal steroidogenesis (letrozole, testolactone, anastrazole, ketoconazole) and antagonizing the peripheral effects of testosterone and dihydrotestosterone (bicalutamide, cyproterone acetate, spironolactone, flutamide) [12].

The inclusion of a LH-RH analogue in the therapeutic schedule should be considered only after recording a spontaneous activation of pituitary-gonadal axis function [16, 17].

Such a treatment strategy seems to be safe and sufficiently effective, at least in terms of reduction of androgenization and deceleration of both growth and bone maturation [9, 11, 16–19]. More numerous and long-term studies, however, are needed to compare the efficacy and safety of the different treatment schedules [12].

## Conclusions

From the analysis of the few available reports on the phenotypic peculiarities of pubertal development in boys with MAS it emerges that: 1) PPP is significantly more infrequent in boys than in girls; 2) the most common testicular abnormality at MAS presentation is macroorchidism, that may be either monolateral or bilateral; 3) macroorchidism is not always associated with clinical and biochemical evidence of PPP; 4) testicular microlithiasis is distinctly more frequent in boys with MAS than in those without MAS; 5) the available therapeutic schedules have to be adopted already at MAS presentation only in the cases with PPP.

## Abbreviations

MAS: McCune-Albright syndrome
PPP: peripheral precocious puberty
TM: testicular microlithiasis
US: ultrasonographic
